# Short and long-term follow-up and clinical outcomes in patients with celiac disease in a large private practice setting

**DOI:** 10.1186/s12876-023-02643-4

**Published:** 2023-01-11

**Authors:** Akash Khurana, Daniel A. Leffler, Kayeromi Gomez, Chandrashekhar Thukral

**Affiliations:** 1 Department of Medicine, University Hospitals/Cleveland Medical Center, 11100 Euclid Ave, Cleveland, OH 44107 USA; 2 Case Western Reserve University, Cleveland, USA; 3 University of Illinois College of Medicine - Rockford, Rockford, USA; 4 The Celiac Center at Beth Israel Deaconess Medical Center, Boston, USA; 5 Harvard Medical School, Boston, USA; 6 Takeda Pharmaceuticals, Boston, USA; 7 Rockford Gastroenterology Associates, Ltd., Rockford, USA

**Keywords:** Celiac disease, Micronutrient, Follow-up, Dietitian, Private practice, Guidelines

## Abstract

**Background:**

Celiac disease (CD) is caused by an immune response to gluten and treatment is adherence to a gluten-free diet. Guidelines from studies in large academic settings recommend registered dietitian (RD) referrals at time of diagnosis and periodic testing for micronutrient deficiencies. There is limited data to guide follow-up parameters in a large, community-based practice. The purpose of this study was to evaluate guideline adherence in this setting.

**Methods:**

This retrospective study conducted in 2019 assessed CD care based on follow-up rates, micronutrient testing, symptoms, and serology results in cohorts with and without RD referrals. Patients in this study were followed at Rockford Gastroenterology Associates (RGA): a large, private GI practice. Patients were included if they had a diagnosis of CD from 1/2014 through 12/2018, based on positive serology and/or duodenal biopsy. Patient data was collected by chart review and analyzed through Microsoft Excel. Fisher’s exact and Chi-square tests were used for the statistical analysis and were calculated through the Statistical Product and Service Solutions (SPSS) software.

**Results:**

320 patients were initially reviewed and a cohort of 126 patients met inclusion criteria. 69.8% had a RD referral. 65.9% had at least one lab test order for any of the 6 micronutrients. Of 63 patients tested for iron, 11 were iron deficient (8 with RD referral). Of 64 patients tested for vitamin D, 21 were deficient (17 with referral). 80.2% attended at least one follow-up appointment, but 34.9% had only one follow-up visit over a mean follow up duration of 5.82 months. 79 patients had follow-up data for symptoms or serology and were separated into 4 categories (with vs. without RD referral): (1) asymptomatic and negative serology (32% vs. 26%), (2) symptomatic and negative serology (28% vs. 16%), (3) asymptomatic and positive serology (27% vs. 32%), (4) symptomatic and positive serology (13% vs. 26%). Category 1 yielded a fisher exact test value of 2.62 (*p* = 0.466).

**Conclusions:**

RD referral, micronutrient testing, and close follow-up are important parameters that affect outcomes in patients with CD. Rates for dietitian referral, some micronutrient testing and follow-up visits were higher than 50%, though results from this study were not statistically significant. Further standardization of follow-up testing and monitoring for CD will help minimize discrepancies between community-based and large, academic GI practices.

**Supplementary Information:**

The online version contains supplementary material available at 10.1186/s12876-023-02643-4.

## Background

CD is a chronic immune-mediated gastrointestinal illness that affects ~ 1% of patients in the United States [1]. CD is triggered by consumption of foods containing gluten (a protein in wheat, rye, and barley) and the immune response produces antibodies against tissue transglutaminase (tTG). The result is an inflammatory reaction caused by cytokines in response to deamidated gluten molecules, which leads to villous atrophy in the small intestine [2]. Patients may present with a variety of symptoms including abdominal pain, diarrhea, steatorrhea, fatigue, weight loss and bloating. [3]

Diagnostic evaluation for CD patients includes screen with serology testing for IgA anti-tTG antibodies, IgG anti-tTG antibodies with serum IgA levels (if a patient is suspected to be IgA deficient) and confirmation with duodenal biopsy with histological analysis, which is the gold standard test for CD diagnosis. To confirm a diagnosis of CD, patients must be on a normal diet prior to serology testing. Further evaluation of CD may include repeat endoscopic evaluation for tissue analysis, repeat serology for antibody detection, and bone mineral density (BMD) testing. [4]

Current treatment for CD is strict life-long adherence to a GFD, which requires effective patient education, individual motivation, and frequent follow-up visits. Despite patients recognizing its importance, adherence to a strict GFD poses a great challenge for many people with CD [5]. Patients with poor adherence to a GFD have reported lower scores on quality-of-life assessments [6] and may also have an increased risk of mortality [7]. Thus, in order to improve the quality of life and survival of CD patients, a thorough review of both initial and repeat diagnostic tests, patient outcomes, follow-up visits, and adherence to treatment recommendations should be considered. Guidelines suggest that patients should be regularly checked for nutritional deficiencies including iron, vitamin D, copper, zinc, folate, and vitamin B12, in addition to being referred to a dietician at time of diagnosis. [4, 8, 9]

Published studies analyzing CD progression are based in large academic settings [6, 10–12]. There is little data on practice parameters and clinical outcomes for CD in a private practice, community-based setting [13]. The aim of this retrospective study was to determine guideline adherence in terms of dietician referral and regular follow-up by gastroenterologists in a large private practice who take care of CD patients in the community, as opposed to a larger academic center.

## Methods

### Study setting and patients

**Fig. 1:**
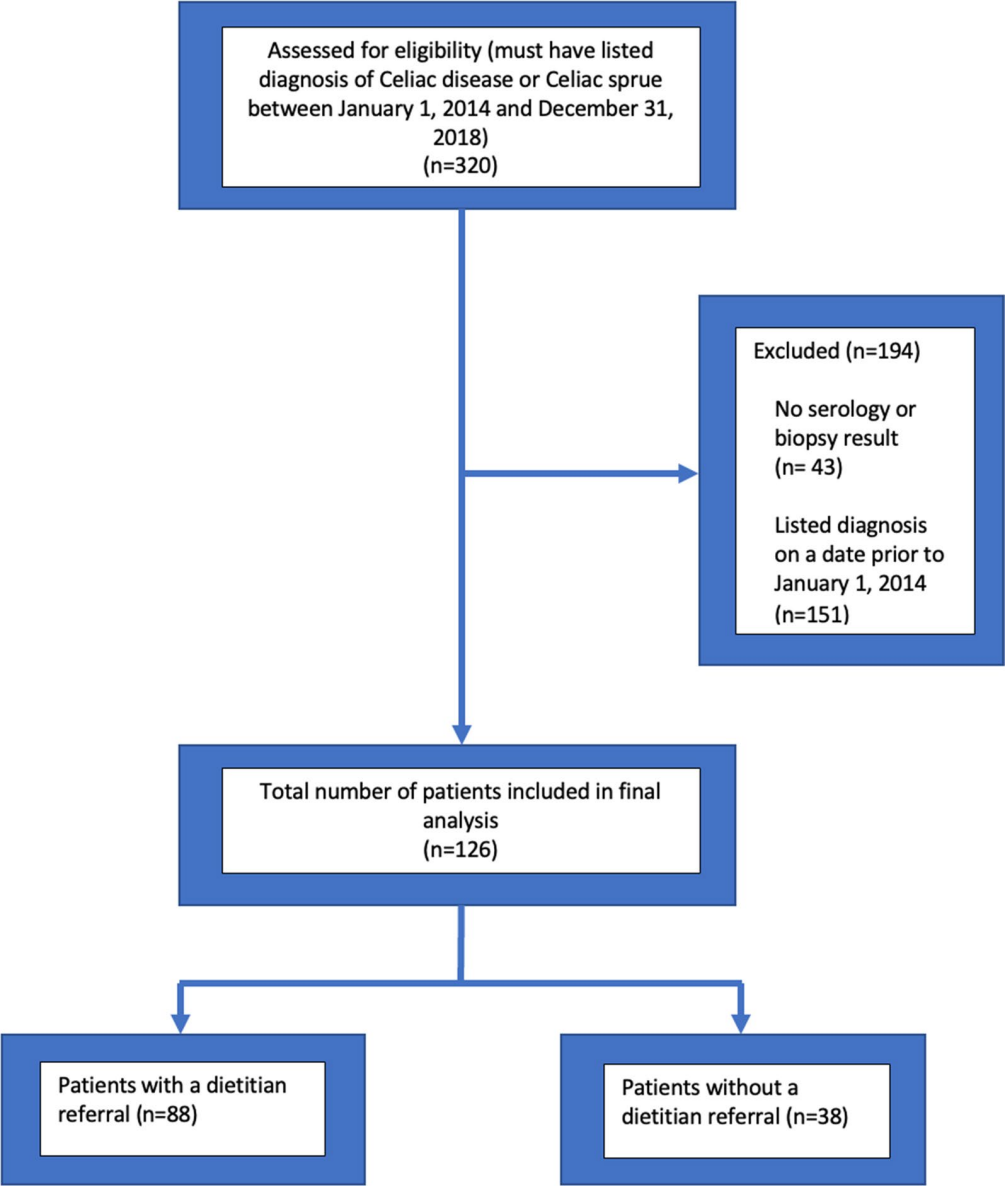
Consort diagram detailing steps taken for inclusion in this study

This study was conducted in 2019 and patients diagnosed with CD that were followed at Rockford Gastroenterology Associates between January 1st, 2014, and December 31st, 2018, were analyzed. Inclusion criteria: (1) patients must be diagnosed with CD based on positive serologic testing and/or duodenal biopsy and (2) patients must be established at Rockford Gastroenterology Associates. Exclusion criteria: (1) patients who did not have a definitive diagnosis of CD based on diagnostic parameters including serology and/or biopsy evaluation. (Fig. 1).

### Data extraction and database creation

Patient data was collected from medical records at RGA. Demographic information, including age, gender, ethnicity, family history of CD and past medical history of autoimmune disease were documented. Initial and follow-up symptoms were recorded if documented. Data was gathered regarding referral rates to a RD at the time of diagnosis, anti-tTG antibody serology results (negative serology: 0–3 U/ml, positive serology: > 4 U/ml), small intestinal biopsy/histology results, and frequency of follow-up visits. We analyzed micronutrient ordering and testing rates at diagnosis and follow up, as well as rates of repeat endoscopy at follow up. Finally, patients were categorized into four categories based on follow-up symptoms and serology: (1) Asymptomatic and negative tTG serology, (2) Symptomatic and negative tTG serology, (3) Asymptomatic and positive tTG serology, (4) Symptomatic and positive tTG serology. [14]

### Statistical analysis

Patient data was analyzed through SPSS and these variables included demographic characteristics, diagnostic findings, reported follow-up visits, and level of repeat testing (if any). Analysis was primarily descriptive and rates of follow-up and response to the GFD among these patients were compared to published literature from large academic centers. In addition, the relationship between symptomatic or histologic response to treatment and the rate of clinical and endoscopic follow was assessed. The fishers exact test and Chi-square tests were used for categorical variables and were calculated through the Statistical Product and Service Solutions (SPSS) software, which is created by International Business Solutions (IBM). A Chi-square test was used only if null hypothesis expected values were greater than 5.

## Results

### Patients

From a total of 320 patients initially identified from the EMR at RGA, a total of 126 patients were confirmed to be diagnosed between January 1, 2014, and December 31, 2018, and met inclusion criteria for this study. In this cohort, 84 (66.7%) were female, 118 (93.7%) were between the ages of 26 and 80, 117 (92.9%) were Caucasian, 23 (18.3%) reported a history of autoimmune disease (Rheumatoid arthritis, Hashimoto’s thyroiditis, Systemic lupus erythematous, etc.) and 16 (12.7%) reported a family history of CD. The baseline characteristics are detailed in Table 1.

**Table 1:** Patient characteristics

Subject characteristics	Number of subjects (n = 126)
*Gender*	
Male	42
Female	84
*Age (years)*	
18–25	4
26–40	37
41–60	44
61–80	37
81 +	4
*History of autoimmune disease*	
Yes	23
No	103
*Family history of celiac disease*	
Yes	16
No	110
*Race/ethnicity*	
Caucasian	117
Hispanic	7
Other	2

### Dietitian referral

A total of 88 (69.8%) patients had a documented referral to a dietitian.

### Follow-up visits

**Fig. 2:**
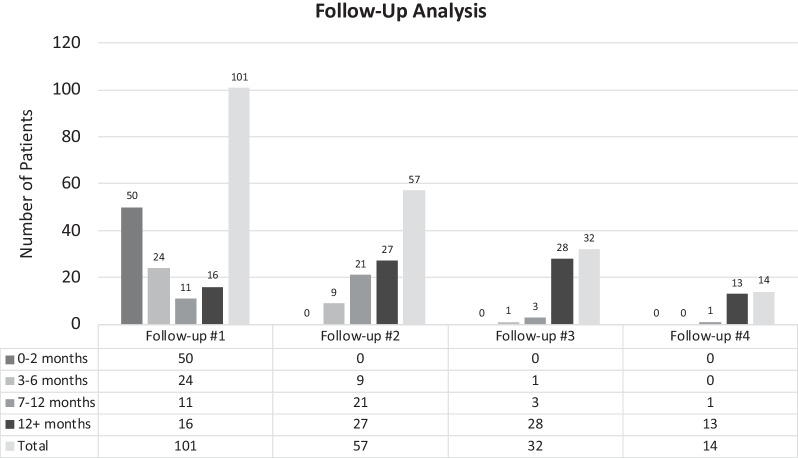
Follow-up visit categorization at chronological time intervals

One hundred and one (80.2%) patients attended at least one follow-up appointment and 57 patients (45.2%) had at least two (Fig. 2). In this cohort, 73 (52.9%) patients were also referred to a dietitian. Of the 80.2% that attended at least one follow-up appointment, 43.6% had only one with 50.5% occurring at 3 or more months after diagnosis. For the first follow-up visit, the average and median time interval after initial diagnosis were 5.82 months and 3 months, respectively. (Additional file 1: Fig. S2).

### Initial and follow-up symptoms

Common symptoms of CD include abdominal pain, bloating, flatulence, diarrhea, weight loss, nausea, and fatigue. During the initial visit these symptoms were endorses by 48, 32, 9, 62, 32, and 26 patients, respectively. At follow-up visits, more than 50% of patients did not endorses the same symptoms, which was consistent across all these symptoms. Additionally, in those with at least one follow-up visit (n = 101), about 79.2% of patients did not report abdominal pain and 100% did not report fatigue, with all other lack of symptom rates in between these two. Patients were divided into four groups based on presence or absence of symptoms and referral to a dietitian (Additional file 1: Tables S1 and S2 and Fig. S1). Data regarding initial and follow-up symptoms does not include data for those without a follow-up visit to record symptoms.

### Initial and follow-up serology and biopsy

A total of 125 patients had an initial serology order/test with 105 (83.3%) at RGA and 20 (15.9%) at another facility prior to consult. 17 patients had negative initial serology results and 2 patients had normal initial biopsy results. 88 (69.8%) patients did have a repeat lab order for a follow-up serology test. 79 patients had a follow-up serology result in the EMR, 60 (75.0%) of which were referred to a dietitian. 24 of these patients had abnormal serology and 36 had normal serology. (*X*^*2*^ = 1.371, *p* = 0.242) 126 patients had an initial biopsy with histological analysis. (Table 2) 22 patients had a repeat endoscopy, and 21 patients had a repeat biopsy result. 10 patients with both initial and follow-up endoscopy with biopsy showed improvement either from complete or partial changes to normal duodenal mucosa. Other data for follow-up serology and biopsy results are shown in Additional file 1: Tables S3 and S4.

**Table 2:** Initial and follow-up histology and biopsy results

Histology	Number of patients	
	Initial	Follow-up
Normal	2	11
Partial	80	8
Complete	44	2
Total	126	21
Serology		
Negative	17	43
Positive	109	36
Total	126	79

**Table 3:** Micronutrient test results at follow-up

Micronutrients	No dietitian referral (# of patients)	Dietician referral (# of patients)
Iron normal	14	37
Iron deficient	3	8
Vitamin D normal	10	33
Vitamin D deficient	4	17
Copper normal	2	12
Copper deficient	0	0
Zinc normal	3	22
Zinc deficient	0	1
Folate normal	6	31
Folate deficient	1	2
B12 normal	7	34
B12 deficient	1	2

*Data for micronutrients is shown in Table 3, Additional file 1: Tables S5 and S6.

### Micronutrients

#### Iron

Initial iron studies (iron/ferritin) were ordered for 62 patients and follow-up iron studies were ordered for 76 patients. 77 patients were initially tested for iron studies and 63 patients were tested at follow-up. Of those tested initially, 42 (33.3%) had results within normal limits. 20 of the patients with normal initial results were not deficient at follow-up and 20 of the patients that were initially deficient were not deficient at follow-up. 6 patients remained deficient. Of the 62 tested at follow-up, 51 had results within normal limits and 11 patients were deficient. 37 patients had a dietitian referral and were tested but not deficient. 42 total patients were not tested at follow-up. (*X*^*2*^ = 0.017, *p* = 0.992).

#### Vitamin D

Initial Vitamin D studies were ordered for 62 patients and follow-up iron studies were ordered for 76 patients. 73 patients were initially tested for Vitamin D and 64 patients were tested at follow-up. Of those tested initially, 30 (23.8%) had results within normal limits. 14 of the patients with normal initial results were not deficient at follow-up and 15 of the patients that were initially deficient were not deficient at follow-up. 12 patients remained deficient. Of those tested at follow-up, 43 had results within normal limits and 21 were deficient. 62 patients were not tested at follow-up. (*X*^2^ = 4.36, *p* = 0.113).

#### Folate and Vitamin B12

Initial folate studies were ordered for 48 patients and follow-up folate studies were ordered for 44 patients. 53 patients were initially tested for folate and 40 patients were tested at follow-up. Of those tested initially, 49 (38.9%) had results within normal limits. 15 of the patients with normal initial results were not deficient at follow-up. Of those tested at follow-up, 37 had results within normal limits and 3 were deficient. 86 patients were not tested at follow-up. (Fisher’s Exact, two-sided *p* = 0.448).

Initial Vitamin B12 studies were ordered for 54 patients and follow-up Vitamin B12 studies were ordered for 46 patients. 64 patients were initially tested for Vitamin B12, and 44 patients were tested at follow-up. Of those tested initially, 59 (46.8%) had results within normal limits. 22 of the patients with normal initial results were not deficient at follow-up. Of those tested at follow-up, 41 had results within normal limits and 3 were deficient. 82 patients were not tested at follow-up. (Fisher’s Exact, two-sided *p* = 0.461).

#### Copper and zinc

Initial copper studies were ordered for 18 patients and follow-up copper studies were ordered for 14 patients. 15 patients were initially tested for copper and 14 patients were tested at follow-up. Both patients that were initially deficient were not tested at follow-up. Of those tested at follow-up, 14 had results within normal limits. 112 patients were not tested at follow-up. (Fisher’s Exact, two-sided *p* = 0.225).

Initial zinc studies were ordered for 36 patients and follow-up zinc studies were ordered for 30 patients. 33 patients were initially tested for zinc and 26 patients were tested at follow-up. Of those tested initially, 27 (21.4%) had results within normal limits. 2 of the patients with normal initial results were not deficient at follow-up and 3 of the patients that were initially deficient were not deficient at follow-up. Of those tested at follow-up, 22 patients had a dietitian referral and were tested but not deficient, whereas 3 patients without a referral were tested but not deficient. 100 patients were not tested at follow-up. (Fisher’s Exact, two-sided *p* = 1.000).

### Follow-up categories

**Fig. 3:**
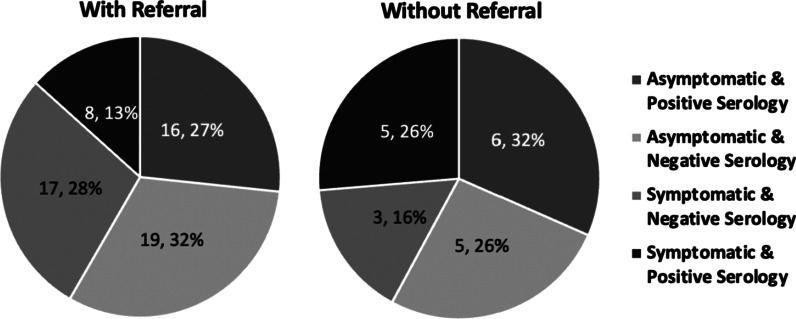
Categorization of patients into four sub-categories

In the cohort of 79 patients that had follow-up data for both symptoms and serology results, 60 also had a referral to a dietitian. 24 patients were asymptomatic with normal serology, 19 of which had a dietitian referral. 13 patients were symptomatic with abnormal serology, 5 of which did not have a referral (Fig. 3) (Fisher’s Exact = 2.62, two-sided *p* = 0.466).

## Discussion

The principal finding of this study was that referral rates to a dietitian was much higher than expected. Providers at RGA ordered iron and Vitamin D studies at greater rates than hypothesized. Testing rates for iron studies were equal to the hypothesized value and Vitamin D studies were greater than hypothesized. However, order and testing rates were lower than the hypothesized values for copper, zinc, folate, and Vitamin B12. Rates of micronutrient deficiencies were low without an identifiable trend when comparing patients with or without a dietitian referral. However, rates of all micronutrient deficiencies declined over time in patients that had both an initial and follow-up value. These trends were most prominently seen for iron and Vitamin D studies, likely because of higher rates of testing (similar trend to results from Deora et al. [15]).

Currently, guidelines set forth by American Gastroenterology Association (AGA) and the National Institute of Health (NIH) recommend periodic follow-up visits at regular time intervals with both a practicing physician and a nutritionist/dietitian [6, 11]. In this study, follow-up visit rates at 3–6 months and beyond were greater than hypothesized. Time intervals between the initial diagnosis and subsequent follow-up visits (if any) were variable and may reflect other gastrointestinal comorbidities or unique circumstances for patients. Patients with a dietitian referral had lower rates of symptomatic disease in comparison to those without a referral. Additionally, patients with a referral had higher rates of negative serology (anti-tTG antibody) at follow-up in comparison to those without a referral.

Although the originally hypothesized number of patients was 320 based on initial polling from the EMR, only 126 met inclusion criteria for the study and the other 194 patient charts lacked significant data for inclusion in the analysis. The smaller number of subjects made it difficult to draw statistically significant conclusions. This discrepancy was emphasized due to an uneven percentage of subjects referred to a dietitian compared to those not referred (69.8% vs. 30.2%),

Other limitations of this study include the following: lack of controlling for other gastrointestinal comorbidities, lack of ability to track a follow-up visit with a dietitian, lack of ability to track patient follow-up with their primary care provider, lack of standardization of national guideline adherence in current practice at RGA, difficulty in standardizing data input procedure regarding patient visits (i.e. symptom data in HPI vs. ROS vs. assessment area, results data in progress notes vs. results section in EMR vs. outside uploaded document), lack of ability to track follow-up for patients diagnosed near the end of the set timeframe (closer to December 2018).

One discrepancy found during the analysis was the difference between micronutrient order rates and actual testing rates. There are several variables that affect these outcomes, many of which are difficult to control for. A future improvement in CD practice and patient outcomes may include artificial intelligence or a mobile application that tracks the variables analyzed in this study. These advances can help providers closely monitor patients in the outpatient setting by mitigating the communication setbacks in the current healthcare system. Providers can monitor dietitian follow-up appointments and GFD adherence with this technology, which may have similar improvement in quality of life and patient outcomes seen with other chronic diseases [16–18].

Currently, most studies analyzing follow-up outcomes for patients with CD have been conducted at larger, academic centers [6, 10–12]. Patients in this study, at a large, community-based gastroenterology practice, showed an improvement in symptomology, biopsy findings, serology findings, and micronutrient deficiencies between the initial visit and most recent follow-up visits. These identified trends, may be due to the strong dietitian referral rates and high rates of medical follow-up, and may highlight underlying themes of strong clinical practice including patient motivation, encouragement, and access to a support network. These are all factors that ultimately help patients with CD adhere appropriately to a GFD.

Although care providers at Rockford Gastroenterology Associates overall showed strong adherence to national guidelines for diagnosis and management of patients with CD, there exists a fair amount of variability in practice patterns. The gastroenterologists at RGA completed training at different, large academic centers at different points in time. Compounding this with differences in clinical judgement, some variability is expected. However, moving forward it is important to improve standardization of management guidelines for clinicians across the board. Further studies, like that conducted by Zanini et al. [13] can help streamline the practice patterns at community based gastroenterology practices in line with academic centers [6, 10–12]. The hope is to improve the overall quality of life for patients with CD by enhancing the comprehensive approach to medical management.

## Conclusions

Registered dietitian referral, micronutrient testing, and close follow-up are important parameters that affect outcomes in patients with CD. In our study, the rates for dietitian referral, some micronutrient testing and follow-up visits were higher than 50%. Although not statistically significant, a greater percentage of patients with a dietitian referral were asymptomatic and/or had negative serology. Additionally, there was large variability in follow-up time intervals and micronutrient lab testing. Further standardization of follow-up and monitoring of CD patients will help minimize the variability between community-based GI practices and academic centers.

## Supplementary Information

**Additional file 1**: **Table S1**. Follow-up symptom analysis in cohorts with and without a dietitian referral. **Table S2**. Follow-up symptom analysis with and without a RD referral. **Table S3**. Repeat biopsy analysis in cohorts with and without a dietitian referral. **Table S4**. Follow-up serology analysis with and without a RD referral. **Table S5**. Follow-up micronutrient study order analysis in cohorts with and without a dietitian referral. **Table S6**. Follow-up micronutrient study testing and deficiency analysis in cohorts with and without a dietitian referral. **Figure S1**. Follow-up symptom results in cohorts with and without a dietitian referral. **Figure S2**. Time interval (in weeks) between initial and follow-up visits

## Data Availability

The database used for this study is available upon request from the authors in the format of a Microsoft Excel file. Authors from this study will be able to electronically deliver the database. Please contact the corresponding author, Dr. Akash Khurana, MD at akhura320@gmail.com.

## Abbreviations

CD: Celiac disease
RD: Registered dietitian
GI: Gastroenterology
RGA: Rockford Gastroenterology Associates, ltd.
GFD: Gluten-free diet
HPI: History of present illness
ROS: Review of systems
EMR: Electronic medical record
tTG: Tissue transglutaminase
BMD: Bone mineral density
