# Supplementary material for: Short and long-term follow-up and clinical outcomes in patients with celiac disease in a large private practice setting
Source: BMC Gastroenterol. 2023 Jan 11;23:9. doi: 10.1186/s12876-023-02643-4 (PMC9835330; doi:10.1186/s12876-023-02643-4)
Supplement: Supplementary file 1 — Additional file 1: Table S1. Follow-up symptom analysis in cohorts with and without a dietitian referral. Table S2. Follow-up symptom analysis with and without a RD referral. Table S3. Repeat biopsy analysis in cohorts with and without a dietitian referral. Table S4. Follow-up serology analysis with and without a RD referral. Table S5. Follow-up micronutrient study order analysis in cohorts with and without a dietitian referral. Table S6. Follow-up micronutrient study testing and deficiency analysis in cohorts with and without a dietitian referral. Figure S1. Follow-up symptom results in cohorts with and without a dietitian referral. Figure S2. Time interval (in weeks) between initial and follow-up visits [file 12876_2023_2643_MOESM1_ESM.pdf]

SUPPLEMENTARY APPENDIX

Symptoms	Dietitian Referral			Statistics		
Frequency<br>Percent<br>Row Pct<br>Col Pct	No	Yes	Total	Chi-Square	p-value	Fisher's Exact Test, two-sided p-value
**Abdominal Pain**				0.9958	0.3183	0.4167
No	24<br>23.76<br>85.71<br>30	56<br>55.45<br>76.71<br>70	80<br>79.21			
Yes	4<br>3.96<br>14.29<br>19.05	17<br>16.83<br>23.29<br>80.95	21<br>20.79			
Total	28<br>27.72	73<br>72.28	101<br>100			
**Bloating**				1.7398	0.1872	0.2763
No	27<br>26.73<br>96.43<br>29.67	64<br>63.37<br>87.67<br>70.33	91<br>90.1			
Yes	1<br>0.99<br>3.57<br>10	9<br>8.91<br>12.33<br>90	10<br>9.9			
Total	28<br>27.72	73<br>72.28	101<br>100			
**Flatulence**				0.0486	0.8256	1.0000
No	27<br>26.73<br>96.43<br>27.55	71<br>70.3<br>97.26<br>72.45	98<br>97.03			
Yes	1<br>0.99<br>3.57<br>33.33	2<br>1.98<br>2.74<br>66.67	3<br>2.97			
Total	28<br>27.72	73<br>72.28	101<br>100			
**Diarrhea**				0.0231	0.8791	1.0000
No	23<br>22.77<br>82.14<br>28.05	59<br>58.42<br>80.82<br>71.95	82<br>81.19			
Yes	5<br>4.95<br>17.86<br>26.32	14<br>13.86<br>19.18<br>73.68	19<br>18.81			
Total	28<br>27.72	73<br>72.28	101<br>100			

Symptoms	Dietitian Referral			Statistics		
Frequency<br>Percent<br>Row Pct<br>Col Pct	No	Yes	Total	Chi-Square	p-value	Fisher's Exact Test, two-sided p-value
**Weight loss**				0.8598	0.3538	0.3928
No	25<br>24.75<br>89.29<br>26.6	69<br>68.32<br>94.52<br>73.4	94<br>93.07			
Yes	3<br>2.97<br>10.71<br>42.86	4<br>3.96<br>5.48<br>57.14	7<br>6.93			
Total	28<br>27.72	73<br>72.28	101<br>100			
**Nausea**				0.0154	0.9012	1.0000
No	27<br>26.73<br>96.43<br>27.84	70<br>69.31<br>95.89<br>72.16	97<br>96.04			
Yes	1<br>0.99<br>3.57<br>25	3<br>2.97<br>4.11<br>75	4<br>3.96			
Total	28<br>27.72	73<br>72.28	101<br>100			
**Anemia/Fatigue**				2.6113	0.1061	0.2788
No	28<br>26.92<br>96.55<br>27.18	75<br>72.12<br>100<br>72.82	103<br>99.04			
Yes	1<br>0.96<br>3.45<br>100	0<br>0<br>0<br>0	1<br>0.96			
Total	29<br>27.88	75<br>72.12	104<br>100			

Table S1. Follow-up symptom analysis in cohorts with and without a dietitian referral

Symptoms	Dietitian Referral			Statistics		
Frequency<br>Percent<br>Row Pct<br>Col Pct	No	Yes	Total	Chi-Square	p-value	Fisher's Exact Test, two-sided p-value
**Symptomatic**				0.3366	0.5618	0.6481
No	19<br>18.81<br>67.86<br>29.69	45<br>44.55<br>61.64<br>70.31	64<br>63.37			
Yes	9<br>8.91<br>32.14<br>24.32	28<br>27.72<br>38.36<br>75.68	37<br>36.63			
Total	28<br>27.72	73<br>72.28	101<br>100			

Table S2. Follow-up symptom analysis with and without a RD referral

Biopsy	Dietitian Referral			Statistics		
Frequency<br>Percent<br>Row Pct<br>Col Pct	No	Yes	Total	Chi-Square	p-value	Fisher's Exact Test, two-sided p-value
**Repeat Biopsy**				0.1206	0.7284	0.796
No	31<br>24.6<br>81.58<br>29.52	74<br>58.73<br>84.09<br>70.48	105<br>83.33			
Yes	7<br>5.56<br>18.42<br>33.33	14<br>11.11<br>15.91<br>66.67	21<br>16.67			
Total	38<br>30.16	88<br>69.84	126<br>100			

Table S3. Repeat biopsy analysis in cohorts with and without a dietitian referral

Serology	Dietitian Referral			Statistics		
Frequency<br>Percent<br>Row Pct<br>Col Pct	No	Yes	Total	Chi-Square	p-value	Fisher's Exact Test, two-sided p-value
**Serology**				1.3714	0.2416	0.3013
Abnormal	11<br>13.75<br>55<br>31.43	24<br>30<br>40<br>68.57	35<br>43.75			
Normal	9<br>11.25<br>45<br>20	36<br>45.00<br>60.00<br>80.00	45<br>56.25			
Total	20<br>25.00	60<br>75.00	80<br>100			

Table S4. Follow-up serology analysis with and without a RD referral

Micronutrient Orders	Dietitian Referral			Statistics		
Frequency<br>Percent<br>Row Pct<br>Col Pct	No	Yes	Total	Chi-Square	p-value	Fisher's Exact Test, two-sided p-value
**Iron studies**				4.1019	0.0428	0.0526
No	23	36	59			
	18.25	28.57	46.83			
	60.53	40.91				
	38.98	61.02				
Yes	15	52	67			
	11.9	41.27	53.17			
	39.47	59.09				
	22.39	77.61				
Total	38	88	126			
	30.16	69.84	100			
**Vitamin D studies**				9.876	0.0017	0.0027
No	23	27	50			
	18.25	21.43	39.68			
	60.53	30.68				
	46	54				
Yes	15	61	76			
	11.9	48.41	60.32			
	39.47	69.32				
	19.74	80.26				
Total	38	88	126			
	30.16	69.84	100			
**Copper studies**				1.884	0.1699	0.2254
No	36	76	112			
	28.57	60.32	88.89			
	94.74	86.36				
	32.14	67.86				
Yes	2	12	14			
	1.59	9.52	11.11			
	5.26	13.64				
	14.29	85.71				
Total	38	88	126			
	30.16	69.84	100			

Micronutrient Orders	Dietitian Referral			Statistics		
Frequency<br>Percent<br>Row Pct<br>Col Pct	No	Yes	Total	Chi-Square	p-value	Fisher's Exact Test, two-sided p-value
**Zinc studies**				7.5966	0.0058	0.0058
No	35	61	96			
	27.78	48.41	76.19			
	92.11	69.32				
	36.46	63.54				
Yes	3	27	30			
	2.38	21.43	23.81			
	7.89	30.68				
	10	90				
Total	38	88	126			
	30.16	69.84	100			
**Folate studies**				4.6044	0.0319	0.0415
No	30	52	82			
	23.81	41.27	65.08			
	78.95	59.09				
	36.59	63.41				
Yes	8	36	44			
	6.35	28.57	34.92			
	21.05	40.91				
	18.18	81.82				
Total	38	88	126			
	30.16	69.84	100			
**Vitamin B12 studies**				5.6069	0.0179	0.0258
No	30	50	80			
	23.81	39.68	63.49			
	78.95	56.82				
	37.5	62.5				
Yes	8	38	46			
	6.35	30.16	36.51			
	21.05	43.18				
	17.39	82.61				
Total	38	88	126			
	30.16	69.84	100			

Table S5. Follow-up micronutrient study order analysis in cohorts with and without a dietitian referral

Micronutrient Results	Dietitian Referral			Statistics		
Frequency<br>Percent<br>Row Pct<br>Col Pct	No	Yes	Total	Chi-Square	p-value	Fisher's Exact Test, two-sided p-value
**Iron**				0.0167	0.9917	
Not Tested	12	30	42			
	11.54	28.85	40.38			
	41.38	40				
	28.57	71.43				
Tested, not deficient	14	37	51			
	13.46	35.58	49.04			
	48.28	49.33				
	27.45	72.55				
Tested, deficient	3	8	11			
	2.88	7.69	10.58			
	10.34	10.67				
	27.27	72.73				
Total	29	75	104			
	27.88	72.12	100			
**Vitamin D**				4.3559	0.1133	
Not Tested	24	38	62			
	19.05	30.16	49.21			
	63.16	43.18				
	38.71	61.29				
Tested, not deficient	10	33	43			
	7.94	26.19	34.13			
	26.32	37.5				
	23.26	76.74				
Tested, deficient	4	17	21			
	3.17	13.49	16.67			
	10.53	19.32				
	19.05	80.95				
Total	38	88	126			
	30.16	69.84	100			
**Copper**				1.884	0.1699	0.2254
Not Tested	36	76	112			
	28.57	60.32	88.89			
	94.74	86.36				
	32.14	67.86				
Tested, not deficient	2	12	14			
	1.59	9.52	11.11			
	5.26	13.64				
	14.29	85.71				
Tested, deficient	0	0	0			
	0.00	0.00	0.00			
	0.00	0.00				
	0.00	0.00				
Total	38	88	126			
	30.16	69.84	100			

Micronutrient Results	Dietitian Referral			Statistics		
Frequency<br>Percent<br>Row Pct<br>Col Pct	No	Yes	Total	Chi-Square	p-value	Fisher's Exact Test, two-sided p-value
**Zinc**				0.1357	0.7126	1.0000
Not Tested	0	0	0			
	0.00	0.00	0.00			
	0.00	0.00				
	0.00	0.00				
Tested, not deficient	3	22	25			
	11.54	84.62	96.15			
	100	95.65				
	12.00	88.00				
Tested, deficient	0	1	1			
	0	3.85	3.85			
	0	4.35				
	0	100				
Total	3	23	26			
	11.54	88.46	100			
**Folate**				0.5632	0.453	0.4478
Not Tested	0	0	0			
	0.00	0.00	0.00			
	0.00	0.00				
	0.00	0.00				
Tested, not deficient	6	31	37			
	15.00	77.50	92.5			
	85.71	93.94				
	16.22	83.78				
Tested, deficient	1	2	3			
	2.50	5.00	7.50			
	14.29	6.06				
	33.33	66.67				
Total	7	33	40			
	17.50	82.50	100			
**Vitamin B12**				0.4968	0.4809	0.4609
Not Tested	0	0	0			
	0.00	0.00	0.00			
	0.00	0.00				
	0.00	0.00				
Tested, not deficient	7	34	41			
	15.91	77.27	93.18			
	87.5	94.44				
	17.07	82.93				
Tested, deficient	1	2	3			
	2.27	4.55	6.82			
	12.5	5.56				
	33.33	66.67				
Total	8	36	44			
	18.18	81.82	100			

Table S6. Follow-up micronutrient study testing and deficiency analysis in cohorts with and without a dietitian referral

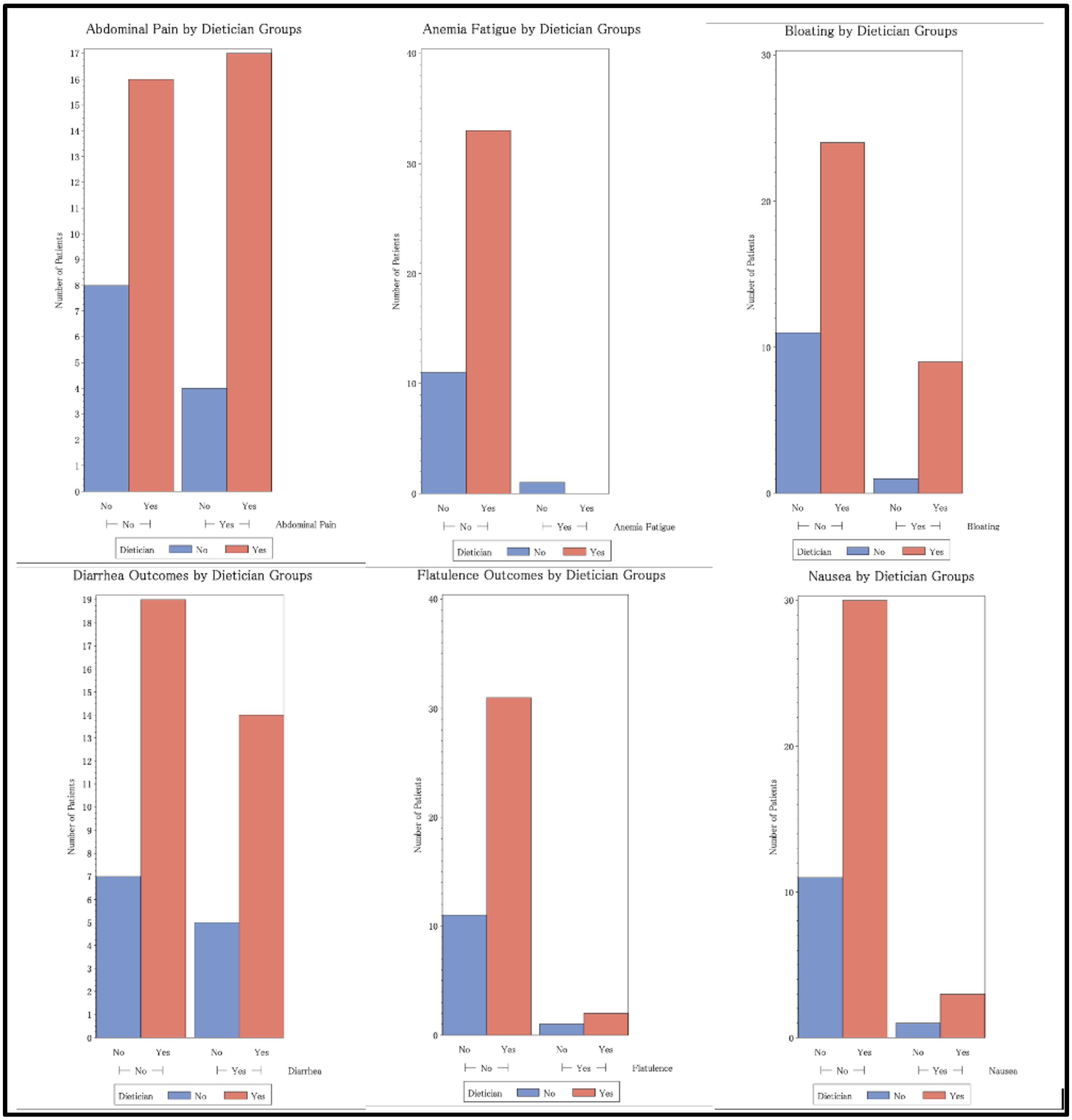

Figure S1. Follow-up symptom results in cohorts with and without a dietitian referral

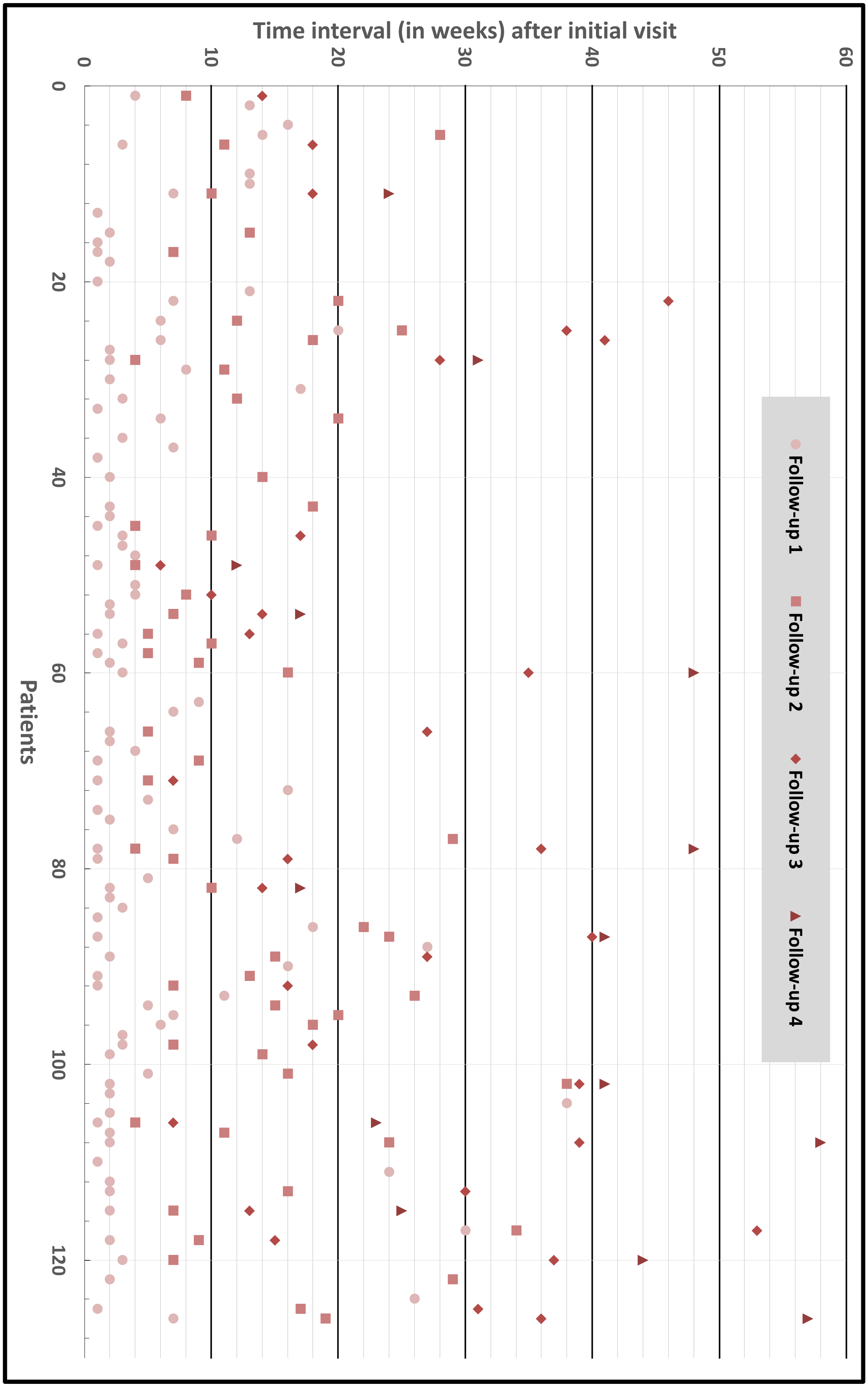

Figure S2. Time interval (in weeks) between initial and follow-up visits
